# 219 Older Adults’ Experiences of a Peer-led Walking Intervention: A Qualitative Study of the ‘Walk with Me’ Randomised Controlled Trial

**DOI:** 10.1093/eurpub/ckae114.091

**Published:** 2024-09-26

**Authors:** Michael Adams, Mark Tully, Liz Simpson, Marie Murphy, Wendy Hardeman

**Affiliations:** Ulster University, Northern Ireland; Ulster University, Northern Ireland; Ulster University, Northern Ireland; Ulster University, Northern Ireland; University of East Anglia, England

## Abstract

**Background and Purpose:**

Development of effective physical activity interventions for older adults is important in eliciting positive health and well-being outcomes. ‘Walk with Me’ is a community-based, peer-led walking intervention aimed at increasing moderate-vigorous physical activity in older adults living in socio-economically disadvantaged areas. This qualitative study aims to understand participant and mentor experiences of the ‘Walk with Me’ intervention.

**Methods:**

Six participant and four peer-mentor focus groups were conducted immediately post-intervention using semi-structured topic guides. Participants and mentors, all aged 60 years or above, were purposely sampled to reflect the wider demographic of the ‘Walk with Me’ intervention. Sessions were audio recorded, transcribed verbatim and validated for accuracy by another author. A framework analysis approach is currently being used for coding transcripts. Analysis will be conducted in the following stages: familiarisation with the audio recordings, coding of the transcripts, development of an analytical framework, application of the framework, charting and interpretation of the data. Analysis will explore the similarities and differences in responses across demographic factors.

**Preliminary results:**

A total of 30 participants (19 females and 11 males) and 16 peer-mentors (nine females and seven males) participated in the focus groups. Each focus group session was comprised of between three and nine individuals, and lasted between 35 and 59 minutes. A preliminary analysis of three participant focus group transcripts suggest that the among the key mediators and moderators of walking in this intervention are social support, feelings of accountability, improved self-efficacy, raised awareness and the physical environment. There are indications that behavioural change techniques of goal setting, feedback and monitoring, problem-solving (overcoming barriers) and action planning may have positively contributed to engagement. Perceived barriers and facilitators to participation, perceived outcomes and perceptions of the implementation of key intervention components have also been provisionally identified.

**Preliminary Conclusions:**

The findings are likely to highlight considerations for implementation, the role of key mechanisms and the contextual factors that may have influenced delivery, receipt and enactment of a peer-led walking intervention. It is expected that findings will help propose refinements to the intervention logic model and recommendations that can inform future studies.

